# From Structure to Function: Mitochondrial Morphology, Motion and Shaping in Vascular Smooth Muscle

**DOI:** 10.1159/000353883

**Published:** 2013-07-24

**Authors:** John G. McCarron, Calum Wilson, Mairi E. Sandison, Marnie L. Olson, John M. Girkin, Christopher Saunter, Susan Chalmers

**Affiliations:** ^a^Strathclyde Institute of Pharmacy and Biomedical Sciences, University of Strathclyde, UK; ^b^Department of Biomedical Engineering, University of Strathclyde Wolfson Centre, Glasgow, UK; ^c^Centre for Advanced Instrumentation, Department of Physics, Durham University, Durham, UK

**Keywords:** Mitochondrial dynamics, Smooth muscle proliferation, Mitochondrial fission, Mitochondrial fusion

## Abstract

The diversity of mitochondrial arrangements, which arise from the organelle being static or moving, or fusing and dividing in a dynamically reshaping network, is only beginning to be appreciated. While significant progress has been made in understanding the proteins that reorganise mitochondria, the physiological significance of the various arrangements is poorly understood. The lack of understanding may occur partly because mitochondrial morphology is studied most often in cultured cells. The simple anatomy of cultured cells presents an attractive model for visualizing mitochondrial behaviour but contrasts with the complexity of native cells in which elaborate mitochondrial movements and morphologies may not occur. Mitochondrial changes may take place in native cells (in response to stress and proliferation), but over a slow time-course and the cellular function contributed is unclear. To determine the role mitochondrial arrangements play in cell function, a crucial first step is characterisation of the interactions among mitochondrial components. Three aspects of mitochondrial behaviour are described in this review: (1) morphology, (2) motion and (3) rapid shape changes. The proposed physiological roles to which various mitochondrial arrangements contribute and difficulties in interpreting some of the physiological conclusions are also outlined.

## Introduction

Mitochondria control virtually every aspect of cell function by providing a continuous supply of adenosine triphosphate (ATP), modulating Ca^2+^ signalling, influencing reactive oxygen species (ROS) levels and regulating redox control (via glutathione and ROS maintenance). Mitochondria may rapidly change from controlling normal cell function to promoting cell death as the organelles also play a central role in necrosis and apoptosis [1,2,3]. Mitochondrial function is acknowledged to depend on the organelles' structure and the organelles' structure is, in turn, controlled by cell function. From these experimental observations the deduction is made that changes in mitochondrial structure are important to the normal function of cells. Support for this deduction seems to come from the frequent dynamic re-organisations of mitochondria that occur in some normal cells and the morphological changes in the organelle that accompany many human diseases, which include myopathies, diabetes mellitus, liver diseases, neurodegeneration, aging and cancer [4,5,6,7,8]. However, the changes in mitochondrial structure may be secondary to (rather than causing) the alteration in cell performance and precisely how changes in mitochondrial structure influence cell function in health and disease has neither been confirmed nor even demonstrated. Nevertheless, the dynamic re-organisation of mitochondria and changes in organelles' structure must regulate cell function. An understanding of the potential physiological functions to which the changes in mitochondrial arrangement may contribute requires characterisation of the mitochondrial phenotype. In this review the diversity of mitochondrial structures and dynamics is outlined along with proposed physiological roles of the various arrangements.

## Mitochondrial Morphologies

Mitochondria have a double membrane arrangement which separates the organelle into four distinct compartments – the outer membrane, the intermembrane space, the inner membrane, and the matrix. Each compartment serves different functions. The outer membrane contains a number of porins which permit free diffusion of molecules into the space between the outer and inner membranes. The space between the two membranes (intermembrane space) contains proteins (e.g. cytochrome c) that play major roles in mitochondrial energetics and apoptosis. In contrast to the outer membrane, the inner membrane is highly impermeable and most ions and molecules require transporters to cross. The inner membrane contains a large component (20%) of the total mitochondrial protein composition, amongst which are transporters for carrying proteins into the matrix (e.g. translocase of the inner membrane) and the enzymes of the electron transport chain. The matrix contains most of the enzymes that are responsible for the citric acid cycle reactions.

Beyond the acknowledged membrane arrangement, the morphology, and indeed distribution of the organelle, varies enormously among cell types. The conventional description of mitochondrial structure is derived largely from electron microscopy (EM) studies and characterizes mitochondria as spherical or short rod structures positioned in various parts of the cytoplasm. However, the significant extent to which mitochondria differ between cells is only beginning to be acknowledged. Mitochondria in fibroblasts are usually long filaments (1-10 μm in length with a fairly constant diameter of ∼700 nm), whereas in hepatocytes mitochondria are more uniformly spheres or ovoids [9]. In native vascular smooth muscle, mitochondria are ovoid or rod-shaped organelles [10,11,12,13], whereas in the endothelium a tubular mitochondrial network exists [14]. Even within individual cells, mitochondrial structure varies. In skeletal muscle, mitochondria are ovoid structures and two populations may exist – one positioned close to the sarcolemma, the other embedded among the myofibrils [15]. The subsarcolemma mitochondria are rounder and smaller than those embedded among the myofibrils [16]. In pancreatic acinar cells there are three different regional groups of functionally unconnected mitochondria; one group in the peripheral basolateral region close to the plasma membrane, another around the nucleus and a third positioned in the periphery of the granular region separating the granules from the basolateral area [17]. The three distinct mitochondrial groups may serve various functions as implied by the observation that each group is activated independently by specific types of cytosolic Ca^2+^ signals [17]. In cardiac myocytes there are also three distinct populations: perinuclear, subsarcolemmal and interfibrillar. Mitochondria in the perinuclear region are more rounded in appearance and densely packed than elsewhere [18,19].

Together these observations highlight some of the structural diversity and suggest mitochondria are ovoid or rod-shaped entities positioned in various parts of the cytoplasm, in broad agreement with conventional EM descriptions of mitochondria. While the resolution of EM has provided detailed insights into mitochondrial structure, the disadvantage of EM is that it provides only a snap-shot of the arrangement of mitochondria at a particular fixed point in time. In addition, EM sections are so thin that the entire mitochondrial morphology cannot be understood without serial sections and image reconstruction, which is rarely done [16,20].

More recently, understanding of the structure of mitochondria has been revised with live cell imaging and the proposal that mitochondria exist only as ovoid solitary structures has been challenged [21]. Rather than being solitary structures, a single mitochondrion consisting of a continuous mitochondrial reticulum spread throughout the cell has been proposed [22,23]. This single structure may permit the rapid diffusion of solutes within the organelle. Two explanations were put forward to explain the solitary ovoid or rod-shaped appearance of the organelle seen in other investigations. First, the solitary appearance may arise from the mitochondria that have fragmented in cells that are unhealthy and oxidatively stressed [24]. Or, secondly, the solitary ovoid-shaped appearance may be image slices of the same mitochondrion sectioned through small slivers of a single mitochondrial reticulum network [21]. The ovoid-shaped mitochondrial structures that have been isolated from cells, and which would appear to support the conventional EM description of mitochondrial structure, were suggested to be a fractionation artefact arising from vesicles fragmented from the original mitochondrial network present within the cell [24].

**Fig. 1:**
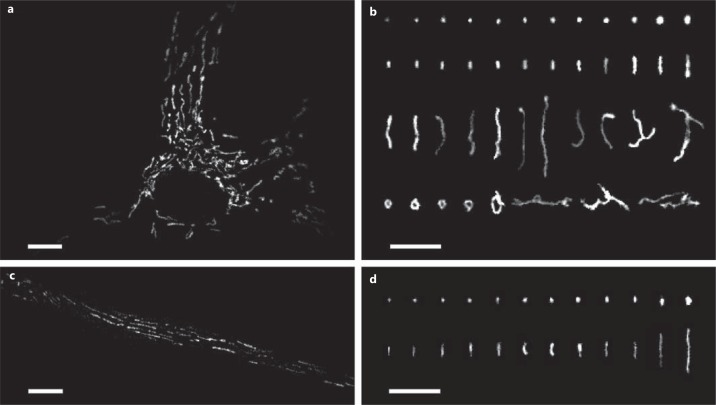
Mitochondrial phenotypes in native and cultured smooth muscle cells. **a** A cultured single vascular smooth muscle cell showing the arrangement of mitochondria. The organelle is scattered through the cytoplasm and is arranged in various orientations. Mitochondria were labelled with MitoTracker Green. **b** Example mitochondria showing the diverse phenotypes that include small spheres, swollen spheres, straight rods, twisted rods, branched rods and loops. **c** A native smooth muscle cell showing the arrangement of mitochondria. The organelle is distributed throughout the cytoplasm and appears to be largely organised parallel to the long axis of the cell. Mitochondria were labelled with MitoTracker Green. **d** Example mitochondria showing the relatively uniform mitochondrial phenotype (when compared to the cultured cell) of spheres and straight rods. Scale bars = 10 μm.

In keeping with the proposal that mitochondria are interconnected structures, in various cultured cells the organelle has a diverse appearance. In cultured vascular smooth muscle, mitochondria exist as long filamentous entities, loops and networks (fig. 1). Several recurring types of mitochondrial structural classes have been identified in cultured cells (fig. 1). These include small spheres, swollen spheres, straight rods, twisted rods, branched rods and loops [25] (fig. 1).

**Fig. 2:**
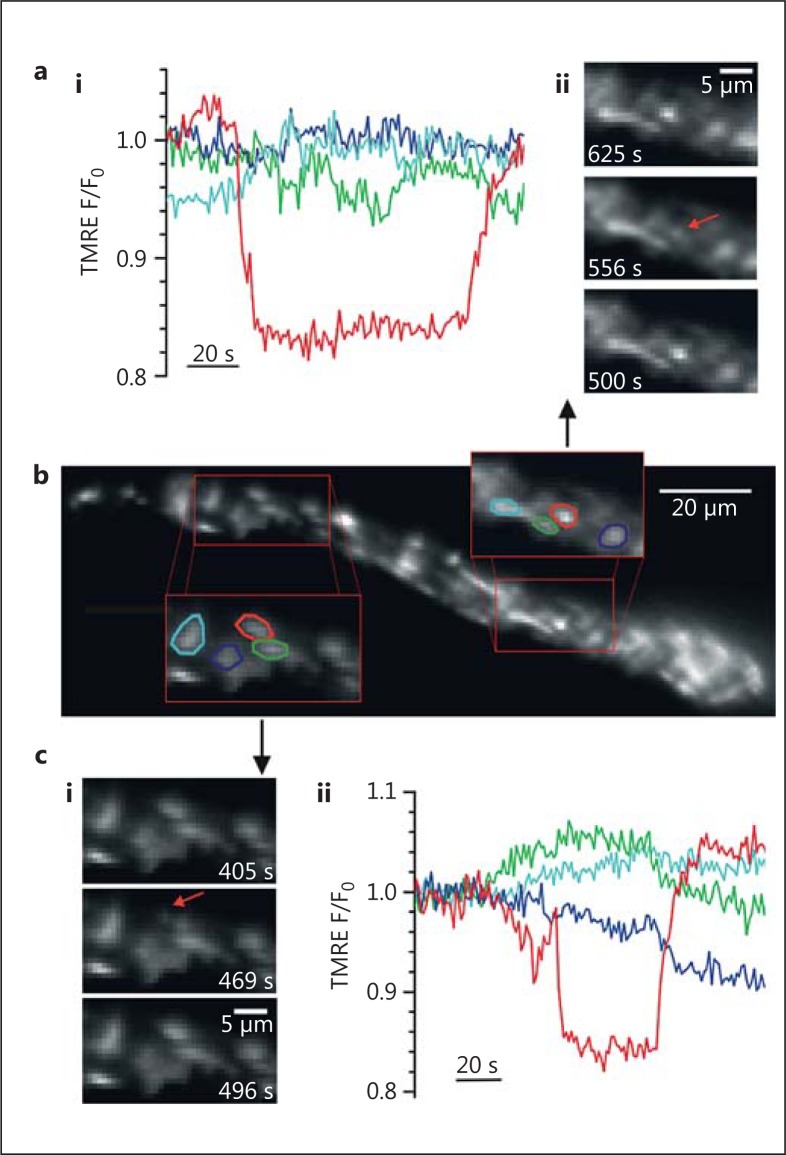
**b** Transient ΔΨ_M_ depolarization in individual mitochondria. The ΔΨ_M_ of individual mitochondria for approximately half of one intact smooth muscle cell. The ΔΨ_M_ was measured by membrane potential fluorophore TMRE (10 nM). The fluorescence intensity from TMRE (10 nM) is directly proportional to the ΔΨ_M_ of individual mitochondria. Two subregions (**a**, **c**) are shown on an enlarged scale and the fluorescence intensity of four individual, neighbouring mitochondria measured (regions are shown circled in four colours that correspond to the four coloured traces in graphs **ai** and **cii**). **aii**, **ci** Selected frames at the times indicated show localized regions of TMRE fluorescence fluctuation (red arrows). **ai**, **cii** Fluorescence intensity (F) of individual regions of interest of corresponding colour, normalized to initial fluorescence values (F_0_) show that in both cases the regions circled in red transiently lose (depolarize) then regain (repolarize) fluorescence. Thus, since the ΔΨ_M_ of mitochondria may change independently of close neighbours, the organelles are a series of individual structures [from Avlonitis et al., [29]].

In native vascular smooth muscle cells, however, mitochondria do not have the same diversity and appear as solitary spheres and rods of various sizes (fig. 1). Indeed, rods and spheres appear to characterize the structure of mitochondria in native cells of most tissues [26]. It seems unlikely that the solitary rods and spheres are a consequence of either oxidative stress or the method of visualizing the organelle. Mitochondria in native cells are not coupled electrically, as would be expected if the organelles formed a continuous network [27,28]. Rather, the membrane potential of individual mitochondria change independently of even very close neighbours (fig. 2) [28,29,30,31,32]. This lack of electrical coupling suggests mitochondria matrices are structurally separate entities. Since the differences in mitochondrial arrangements in cultured and native cells were measured under identical imaging conditions (fig. 1), the organelle may undergo a reorganisation in culture conditions. Notwithstanding the apparently less complicated appearance, there are a wide variety of mitochondrial sizes in native vascular smooth muscle cells (∼0.5-10 μm; fig. 1). Whether or not the larger structures are single large mitochondria, several clustered but separate mitochondria or a localized network is presently unknown. Indeed, although to date changes in mitochondrial morphology have not been directly observed, they may occur in native muscle cells. For example, in the vascular system in pulmonary hypertension [33], mitochondria were smaller than those in control pulmonary artery smooth muscle. In venous smooth muscle from patients with renal failure [34], mitochondria were larger than controls. These observations suggest the organelle's structure undergoes change in vascular disease. Other changes occur. Giant mitochondria are seen in aging cells and in cells with metabolic injuries [5,6]. In atrophied skeletal muscle, as a result of autophagy, the distribution of mitochondria changes from an organised arrangement of intermyofibrillar and subsarcolemmal mitochondria, to scattered and disorganized throughout the fibre [35]. Together, despite the relatively constant appearance of mitochondria in native cells, structural reorganisation does occur at least in cases of stress.

### Functional Significance of the Various Mitochondrial Morphologies

While the significant differences in the structure of mitochondria are presumably important for cellular physiology, the function(s) served by the various arrangements is unclear. One proposal is that mitochondrial morphology and distribution is determined by the energetic requirements of the cell. Early studies showed that a reversible ultrastructural change in mitochondria accompanied changes in metabolic state. Active mitochondria, from native liver cells, had an increased matrix electron opacity and a smaller matrix volume than metabolically inactive mitochondria [36]. The relationship between small mitochondria and high energetic requirements of the cell was questioned in later studies [37,38]. Mitochondrial networks, it was subsequently proposed, characterize metabolically and energetically active cells and small mitochondria are more common in quiescent and respirationally inactive cells [37,38]. The prevalence of mitochondrial networks in cultured muscle cells, including those derived from heart [37], appeared to support the relationship between the occurrence of networks and energetic activity. The extended interconnected mitochondrial network was suggested to enable efficient mixing of mitochondrial content to increase the respiratory activity [37]. However, the proposed relationship between networks and high metabolic activity was itself questioned by the observation that experimentally increasing the extent of networking *decreased* mitochondrial respiration in HeLa cells [39].

It is of significance that, in mammalian cells, mitochondrial networks appear to be unique to cultured cells, and the proposed relationship between networks and energetically active cells is at odds with mitochondrial morphology in native cardiac and smooth muscle cells. In each of these native, high-energy-demand cell types mitochondria are ovoid or short rod shapes. Perhaps the increased surface area of small mitochondria may facilitate the exchange of metabolites.

Assigning a function to the mitochondrial morphology that exists in a particular cell type (e.g. networks in energetically active cells) has also been complicated by the observation that pronounced mitochondrial morphological changes can occur within one cell type, most notably during cell cycle progression. At the G1-S cell cycle transition, mitochondria change from being isolated, fragmented organelles to a hyperfused network in the normal rat kidney epithelial cell line [38]. The reorganisation may be important for determining cyclin E buildup and cell cycle progression [38].

Perhaps rather than energetic status, changes in mitochondrial structure may influence other crucial but more transient cellular functions such as Ca^2+^ and ROS signalling, or biosynthetic processes. A mitochondrial network, for example, may determine the nature of the mitochondrial Ca^2+^ signal. In HeLa cells stimulated with histamine, mitochondrial Ca^2+^ uptake initiated at preferential points of the mitochondrial network and the [Ca^2+^] increase travelled along the network [40]. When the network was fragmented, local increases in mitochondrial [Ca^2+^] occurred in response to histamine, but diffusion of Ca^2+^ was limited and smaller mitochondria had a smaller Ca^2+^ rise [40]. A mitochondrial network near the nucleus may also be important for gene transcription in response to hypoxia in intact lungs and cultured pulmonary artery endothelial cells [41]. In this case, perinuclear clustering of mitochondria was triggered by hypoxia and resulted in increased ROS in the nucleus, which in turn caused oxidative modification of the vascular endothelial growth factor promoter, decreasing the transcriptional complex assembly and mRNA expression [41].

Thus, in cultured cells there are a wide variety of mitochondrial phenotypes which include small spheres, swollen spheres, straight rods, twisted rods, branched rods and loops, and there are clear changes in mitochondrial organisation that occur between and within cells. In native cells mitochondria morphology changes during stress, but the physiological function and significance of the changing mitochondrial phenotype is largely unknown. The question arises, if mitochondrial networks are critical to normal cellular and mitochondrial function, how do many native cells maintain activity in the virtual absence of a structural mitochondrial network?

## Mitochondrial Motion

**Fig. 3:**
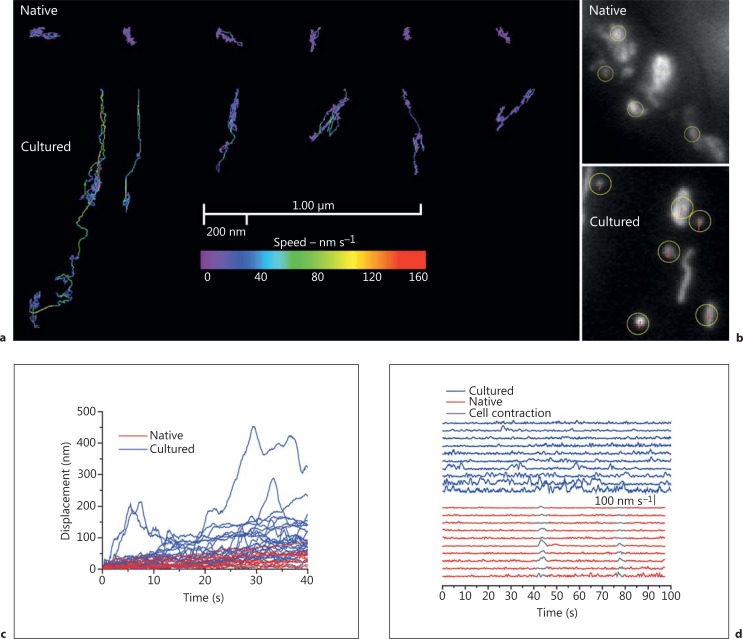
Mitochondria are largely immobile in native and highly dynamic in cultured cerebral resistance artery smooth muscle cells. **a** Motion tracks of the 6 most motile mitochondria from native cells (top) are compared with the organelles' typical movement in cultured cells (bottom). The plot shows the x-y displacement and velocities of each mitochondrion. Mitochondria in native cells show almost no movement, whereas those in cultured cells engage in brief bursts of motion at speeds of ∼160 nm s^-1^, resulting in substantial movement of the organelle. **b** To provide a sense of scale of the movement the motion tracks (in red) are overlaid on the mitochondria images. The yellow circles show the position of the mitochondria's centre in the first frame of the sequence. The red motion tracks on the mitochondria of the native cells are ∼1 pixel and so difficult to visualize. **c** The displacement of the tracked mitochondria plotted as a function of time. Displacement is defined as the distance between the position of an organelle at time t and at time t = 0. Mitochondria in cultured cells (blue) undergo bursts of motion that cover large distances when compared to the displacement of mitochondria within the native cells (red). **d** The instantaneous speed of tracked mitochondria (native, red; cultured, blue) was measured by comparing the organelles position over 1-second intervals. The speeds have been separated in the vertical axis for clarity. Aside from two global motion events due to slight contraction of the entire cell (shown in grey) the mitochondria in the native cell are inactive. In the cultured cell many bursts of high-speed motion occurred with a maximum speed of typically 100 nm s^-1^. In all experiments TMRE was used to visualise mitochondria and the experiments were carried out at room temperature [from Chalmers et al., [26]].

In many cells the distribution of mitochondria is not uniform and the organelles accumulate in particular subcellular regions which require high metabolic activity. Examples include mitochondrial accumulation in active growth cones of developing neurons [42] and dendritic protrusions in spines and synapses [43]. The apparently specific location of mitochondria implies that movement of mitochondria occurs. Indeed, in various cultured cells, including cultured vascular myocytes, mitochondria appear to move almost constantly. Several types of dynamic behaviour have been identified [44,45,46] and include Brownian motion, in which the organelle shows mostly small-scale, thermally driven, random movements (fig. 3). Stochastically determined, directed motion is another type of movement [44] in which short-range motor-driven events occur with various randomly determined durations. Long-range motor-driven displacement is also frequently evident. In this case the organelle travels at significant speeds, at times for considerable distances, in a single path. In our observations, the speed of mitochondria undergoing directed motion was variable and short bursts of motion up to 160 nm s^-1^ occurred at room temperature. At 37°C the movement was significantly faster and bursts reached velocities of 1,000 nm s^-1^. Other types of mitochondrial movement include mitochondrial extension, in which the organelle increases its length by a process which is quite distinct from fusion (online suppl. video 1; for all online suppl. material, see www.karger.com/doi/10.1159/000353883). Retraction is another type of motion in which the mitochondria decreases its length by a process other than fission (online suppl. video 1). Extension and retraction, in our observations, occurred in rod-shaped but not in spherical mitochondria. Rod-shaped mitochondria, when undergoing directed motion, may significantly change shape and the organelle may extend, retract and bend while moving. Spherical mitochondria do not appear to alter their shape when moving.

Motor-driven mitochondrial displacements, including those in vascular smooth muscle, are bidirectional – some organelles move towards, others away from the nucleus. On occasion mitochondria rapidly switch directions, on the timescale of seconds. Mitochondria may also shift between rapid movements with speeds of several hundred nm s^-1^ and being essentially stationary. The various observations suggest that the machinery on the mitochondrial membrane must include motors and anchoring components.

**Fig. 4:**
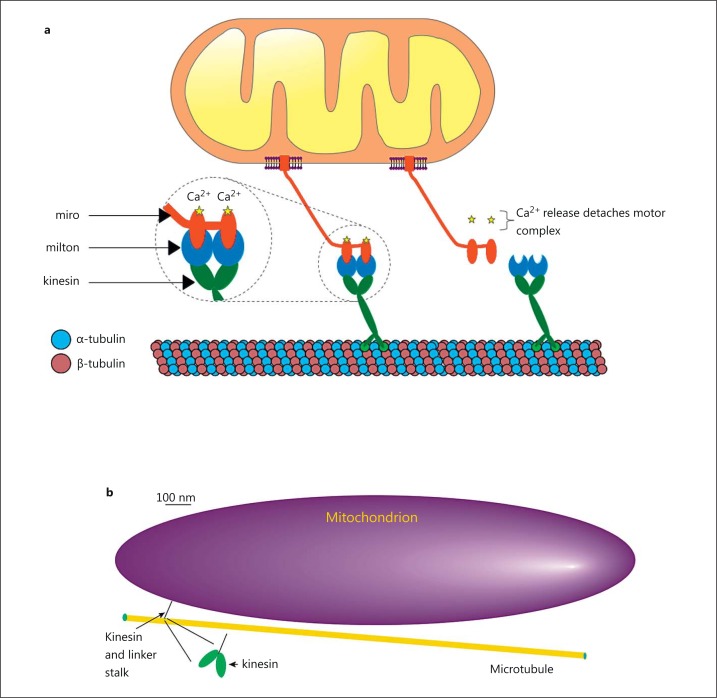
Illustration of the general scheme adopted by the eukaryotic organelle transport network. **a** Tubulin dimers polymerise to form a cylindrical microtubule. One end of the molecular motor (kinesin or dynein; green) binds to sites on the microtubule along which they ‘walk’. The other side of the molecular motor attaches to specific membrane-associated proteins (e.g. miro or syntabulin; red) on the mitochondria via specialized linker proteins (milton; blue) [8]. Signalling molecules, such as Ca^2+^, regulate the attachment (left inset) of the motor/linker complex [7,8] to provide mitochondrial positioning control [108]. **b** Kinesin is vanishingly small when compared to the size of the mitochondrion. The cartoon of a mitochondrion, microtubule and kinesin motor protein is drawn approximately to scale. The microtubule is ∼24 nm in diameter and has attached the kinesin motor protein feet. The feet are separated by ∼5 nm [50]. The kinesin feet are attached to the mitochondrion via a lengthy (∼70 nm) coiled coil connecting stalk [109]. The mitochondrion drawn in the figure is 2,000 × 500 nm. Kinesin is small when compared to the mitochondrion (the inset shows an expanded view of kinesin feet). Perhaps multiple kinesin motors attach to a mitochondrion to achieve higher speeds in mitochondrial movement [51,110]. An interplay of multiple motors moving in opposing directions may create a ‘tug of war’ to regulate the organelle's direction of movement and speed [111,112].

In mammalian cells, the microtubule network provides the backbone on which organelle transport occurs with the help of motor and linker proteins [47] (fig. 4). Immobilization of mitochondria on the cytoskeleton can be achieved by docking with actin [48] or microtubules, the later using syntaphilin [49]. Microtubules are hollow, 24-nm (outer diameter) cylinders built by polymerizing repeating dimer units, each consisting of an alpha- and beta-tubulin globular protein. The motor proteins are the kinesin and dynein families of microtubule-binding ATPases that provide the source of processive motion of mitochondria (fig. 4).

Kinesin and dynein motors each consist of a pair of microtubule-binding globular proteins (feet) that are attached to a long body (fig. 4). The feet bind to specific sites on microtubules and each binding site is ∼8 nm apart. In the case of kinesin, each foot attaches to successive binding sites on the microtubule so kinesin ‘steps’ are each about 8 nm. In our studies, mitochondrial velocities of 1,000 nm s^-1^ we observed in single cells at 37°C [26]. This velocity corresponds to 125 kinesin steps s^-1^, which is close to the maximum speed of the kinesin motor reported from in vitro assays [50]. Dynein ‘steps’ may be larger than those of kinesin; dynein feet are separated by between 1 and 4 binding sites along the microtubules, so dynein steps are 8, 16, 24 or 32 nm apart [51,52].

Significantly, the intrinsic structural polarity of the microtubule ensures that motor feet bind aligned in a specific direction to effectively create a ‘front foot’ and ‘back foot’. ATP hydrolysis triggers the unbinding of motor feet from the microtubule and, by a mechanism that is not fully understood, preferentially unbinds the back foot. Some mix of stochastic Brownian forces and directed force, employing energy from ATP hydrolysis, may move the back foot two binding sites along the microtubule, i.e. to the next available binding site ahead of the previous front foot. The foot binds at the new site, progressing the motor protein centre of mass by one binding site and generating processive motion of the motor and attached mitochondria [53]. The molecular motors are vanishingly small when compared to the size of the cargo (mitochondria) moved (fig. 4b), which emphasizes the enormous power generated by kinesin and dynein. Indeed, the power to weight ratio of kinesin far exceeds that of modern jet engines [50].

In native cell types motor-driven displacement is less frequently observed (fig. 3) and mitochondria may not move at all [26,54]. In the cardiovascular system, particularly little is known of mitochondrial movement in native cells. In adult heart cells and the intact heart, while all the necessary proteins for mitochondrial motion to occur are expressed, current research has been unsuccessful in measuring mitochondrial motion, i.e. there are no reports showing significant mitochondrial movements. The only changes detected in adult heart cells has been a restricted Brownian motion which was attributed to morphological changes of the organelle arising from contraction and expansion of the mitochondrial matrix (condensed/orthodox transitions) [54]. In native vascular smooth muscle cells the great majority of mitochondria show only a restricted Brownian displacement, which does not significantly displace the organelle from its position (fig. 3) [26].

### Functional Significance of Mitochondrial Motion

While significant advances have been made on the identity of proteins mediating and regulating mitochondrial movement, an understanding of the physiological purpose and cellular function of mitochondrial motion is much more preliminary. The function of one type of mitochondrial dynamics, motor-driven displacement, is probably most studied in neurons [8,55,56]. Mitochondria are synthesized in the neuronal cell body [57] and then transported down the axon [58]. Damaged mitochondria, such as those unable to maintain the membrane potential (i.e. depolarised), are, it is proposed, transported back towards the cell body for degradation [46]. This hypothesis is supported by the observation that depolarising mitochondria using the complex III inhibitor antimycin, or complex I inhibitor annonacin, increased mitochondrial movement towards the cell body (retrograde) but had little effect on mitochondrial movement away (anterograde) from the cell body [46,59]. However, there is conflicting data [60]. Mitochondrial depolarisation using the uncouplers CCCP and FCCP blocked all cytoplasmic transport, while another depolarising agent, DNP (also an uncoupler), affected none [61]. Disrupting mitochondrial function by depleting mtDNA, which leads to disruption of the respiratory chain and increased susceptibility to oxidative stress, approximately doubled bidirectional movement of mitochondria (retrograde and anterograde) and mitochondrial density along the proximal axons increased [62]. Together, these observations are not fully compatible with the proposal that compromised neuronal mitochondria are transported for degradation to occur and further study is required.

Another proposal to explain the physiological significance of mitochondrial movement arises from the observation of a dramatic repositioning of the organelle during chemotactic migration in lymphocytes. Mitochondria redistribute to the rear of the moving activated T cells (the uropod) where they are thought to provide ATP to maintain cell motor activities [63]. Mitochondrial movement may also be required to support cell migration in cancer cells. In breast cancer cells, mitochondria appear to move to the front of the cell (leading edge) during directed movement. Experimental manoeuvres that reduced mitochondrial movement (e.g. by generating elongated mitochondria or clusters) suppressed chemoattractant-induced recruitment of mitochondria to lamellipodial regions, inhibited lamellipodia formation and reduced the metastatic abilities of breast cancer cells [64]. Increasing mitochondrial movement (by reducing the size of mitochondria) resulted in more mitochondria in lamellipodia regions, increased lamellipodia formation and enhanced metastatic abilities of breast cancer cells [64].

Motor-driven displacement of mitochondria has been linked frequently to positioning the organelle at locations where there is an energy demand [48,65]. This hypothesis presumably requires that significant ATP gradients occur, although direct evidence for supporting the proposal is lacking. Indeed, in an interesting experiment designed to study ATP gradients, mature mouse oocytes (which rely entirely on mitochondria for ATP production) were centrifuged so that the entire mitochondrial complement was displaced to one side of the oocyte. After centrifugation, when mitochondria were restricted to one side of the oocyte, no [ATP] gradient (measured with luciferase imaging) existed across the cytoplasm between the 50-μm mitochondria-free end of the oocyte and the region which contained mitochondria [Prof. Karl Swann, University of Cardiff, pers. commun.). These results suggest that ATP diffusion was rapid enough to prevent gradients developing in resting cells.

On the other hand, changes in ATP levels throughout the cell (i.e. global [ATP]) may correlate with mitochondria undergoing translocation and aggregation during oocyte maturation [66]. Bursts of global [ATP] increases occurred as mitochondria underwent translocation and aggregated into clusters in the perinuclear region [66]. While the mechanism that links the extent of clustering of mitochondria and the ability of the organelle to generate ATP is not clear [66], these observations none-the-less highlight a physiological consequence of a change in mitochondrial behaviour.

One way mitochondrial positioning to certain parts of the cell may occur is by increases in [Ca^2+^]_c_ inhibiting motor-driven mitochondrial movement [8,67,68,69] to locate the organelle where cell activity is increased (as assessed by Ca^2+^ signals). Mitochondria positioned at these sites may then modulate the signals themselves. The subplasma membrane localization of mitochondria in T lymphocytes and smooth muscle modulated the plasma membrane store-operated ion channel and Na^+^/Ca^2+^ exchanger activity, respectively [70,71], while mitochondria near the sarcoplasmic reticulum [72] exerted a significant control over Ca^2+^ release by modulating the activity of IP_3_ receptor clusters in smooth muscle [73].

The notable absence of movement in native cells contrasted with the pronounced movement in rapidly dividing cultured vascular myocytes (fig. 3) and led us to hypothesize that motion was a requirement for proliferation to occur [26]. We found that mitochondria, in intact resistance arteries, were largely immobile. However, significantly, in a small number of cells in the intact resistance artery, mitochondrial movement occurred. This observation was one of the few direct observations of mitochondrial motion in any intact tissue and remains the only observation in intact tissue in the cardiovascular system. We speculated that cells in which mitochondrial movement occurred may be undergoing proliferation. In support, when proliferation was encouraged in the intact artery in organ culture, the extent of mitochondrial motion in the intact artery increased. When mitochondrial motion was reduced, proliferation was inhibited, as revealed by decreased expression of proliferative markers (in intact arteries) and ^3^H thymidine uptake (in cultured cells) [26]. Fluorescence-activated cell sorting, FACS, was used to examine cell cycle distribution and revealed that suppressing mitochondrial movement increased the fraction of cells in G0/G1 and decreased those in the S phase [26]. Disturbing mitochondrial behaviour may result in mitotic chromosome misalignment during mitosis [38] and activate the G0/G1 checkpoint in the succeeding cell cycle [26]. Together, these observations suggest that mitochondria are adaptable and may switch between being rigidly immobile to being highly mobile entities as vascular smooth muscle switches from native nonproliferative to a proliferative form. This mitochondrial plasticity is an essential mechanism for the development of smooth muscle proliferation.

In a related but separate proposal, mitochondrial movement between cells may trigger proliferation in multipotent mesenchymal cells. When maintained in co-cultures, mitochondria moved from vascular smooth muscle to multipotent mesenchymal cells via tunnelling nanotubes. The mitochondrial movement increased proliferation of multipotent mesenchymal cells [74].

Thus, the precise functions served by mitochondrial movement are not yet clear, though several possibilities exist. Mitochondrial movement may be involved in the organelles' turnover, localized ATP provision or cell proliferation.

## Mitochondrial Shape Changes

Kinesin- and dynein-dependent movement of mitochondria along microtubules requires cargo of a particular size for transport to take place. A mitochondrial network, therefore, must be divided into smaller organelles that can be moved readily by the motors [65]. To this end, the machinery that transports mitochondria is probably coordinated with another type of dynamic behaviour – that of combining (via fusion) or separating (via fission) mitochondria.

Fusion and fission operate on the two lipid bilayers that surround mitochondria and are achieved by several proteins that are sometimes referred to as ‘mitochondria-shaping proteins'. These proteins are dynamin-related guanosine triphosphatases (GTPases) [75] and include the fusion proteins mitofusin (Mfn1 and Mfn2) and optic atrophy 1 protein (Opa1) and the fission proteins, dynamin-related protein 1 (Drp1) and its outer mitochondrial membrane adaptor fission protein 1 (hFis1) [75].

Mfn1 and Mfn2 are located in the outer mitochondrial membrane [76] and are involved in the first step in fusion. Mfn1/2 in one mitochondrion interacts with mitofusins on another to tether adjacent organelles [77]. GTP hydrolysis contributes to the next step in fusing the outer membranes. When GTPase activity is prevented, mitochondria tether but do not fuse [78]. Opa1 is also required for fusion to occur. Opa1 is either in the intermembrane space or bound to the surface of the inner mitochondrial membrane, and is required to tether and fuse the inner membrane of the organelle [79].

Mammalian mitochondria undergo fission by the interaction of the cytosolic protein Drp1 and outer mitochondrial membrane-anchored protein hFis1. Drp1 is recruited from the cytosol by hFis1 to form spirals around mitochondria that constrict to sever both inner and outer membranes. The endoplasmic reticulum (ER) may play an active role in determining the sites of mitochondrial fission. Drp1 assembles on mitochondria at sites of mitochondrial-ER contact to evoke mitochondrial fission at sites close to the ER [80]. At the mitochondria-ER interaction site, another protein, inverted formin 2, may also contribute to fission by acting to polymerize actin to initiate mitochondrial constriction and facilitate Drp1 ring assembly at the constriction site [81].

**Fig. 5:**
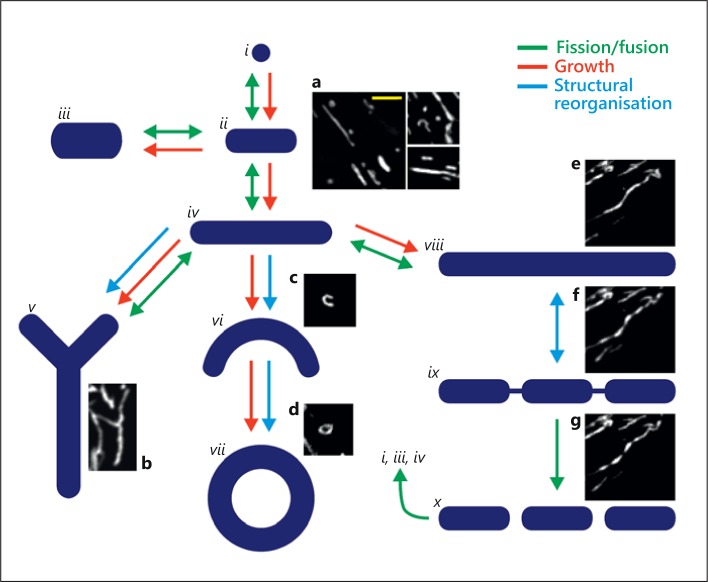
Proposed routes for dynamic changes in mitochondrial morphology. By fission, fusion, growth and structural reorganisation, mitochondria continuously remodel to create a diverse range of morphologies. The diagram structures *(i-x)* and the associated arrows illustrate potential routes of morphological change, alongside representative live-cell images of mitochondria from cells stained with MitoTracker Green (**a-g**). Globular (*i*, **a**) and rod-like structures (*ii-iv*, **a**) can be transformed into both branched (*v*, **b**) and curved structures (*vi-vii*, **c**, **d**), whilst elongated rod-like mitochondria can fragment into multiple smaller mitochondria (*viii-x*, **e-g**). **e-g** Images are taken from a single image series, a movie of which is provided in the supplementary material (online suppl. video 2, ‘sausage string movie’ running at ∼40× real-time speed). The magnification is the same for all images and the yellow scale bar (**a**) = 5 μm.

Fission and fusion interact to create a diversity of mitochondrial structures (fig. 5; online suppl. video 1). The arrangements generated include rods of various lengths, sausage string appearance, looped structures and various branched and network arrangements (fig. 5; online suppl. videos 1 and 2). Fission and fusion are widely observed in cultured cells but are infrequent events in native cells [9]. Nonetheless, some support for the occurrence of fusion, at least, in native cells is found in the EM observation that mitochondria were in contact at points of electron-dense plaques [16]. This observation suggested outer mitochondrial membrane tethering occurred in native skeletal muscle [16]. Evidence for the complete fusion of the organelle in native cells comes from work in mouse skeletal muscle which was modified to express a mitochondrial-targeted photoactivatable green fluorescent protein (GFP) [82]. On photoactivation the matrix-located GFP moved among neighbouring mitochondria, indicating that mitochondrial fusion occurred even though the organelle appears static and well organised [82]. In native cardiac muscle maintained in short-term culture (3 days), a matrix-located fluorophore also moved among neighbouring mitochondria either via transient fusion (‘kiss and run’ [83]) events or transient formation of a connecting nanotubular system [84].

### Functional Significance of Fission and Fusion

In various cardiovascular models (e.g. coronary ligation [85], ischemia reperfusion injury [86,87], pulmonary hypertension [33], diabetes mellitus [14]) mitochondria change to become unusually large or small [88], suggesting that fission or fusion may have occurred (although not directly observed). Significantly, at least in some cases, preventing the changes in mitochondrial shape may be protective. The small-molecule mitochondrial fission inhibitor Mdivi-1 reduced cardiomyocyte cell death after ischemia-reperfusion and reduced myocardial infarct size in vivo in mice subjected to coronary artery occlusion [86]. Mdivi-1 also reduced tubular cell apoptosis and kidney damage during renal ischemia/reperfusion injury [87]. In pulmonary hypertension, inhibiting fission with Mdivi-1 reduced the muscularization of small pulmonary arteries in pulmonary hypertensive animals. This effect decreased pulmonary vascular resistance and right ventricular hypertrophy and increased exercise capacity [33]. This experimental work suggests that mitochondrial reshaping is important in the changes that occur in disease.

Additional support for the importance of fission and fusion to *normal* physiological function is found, it is proposed, from the consequences of disrupting the function of proteins involved in fusion and fission. Mice with mutations in Mfn1, Mfn2 or Opa1 do not survive gestation [89]. In humans, Mfn2 mutations cause defects in peripheral neurons and are associated with Charcot-Marie-Tooth type 2A neuropathy [90], a condition producing motor and sensory deficits. Opa1 is mutated in a neuro-ophthalmic condition (dominant optic atrophy) in which there is degeneration of the optic nerves, causing visual loss [90]. Mutations in proteins contributing to mitochondrial fission can also be lethal. Mice lacking Drp1 do not survive gestation [91] and interfering RNA directed against Drp1 is lethal in *Caenorhabditis elegans* embryos [92].

The diversity and quantity of serious physiological changes which arise from mutations in the proteins controlling mitochondrial fusion and fission are interpreted widely as evidence both for the occurrence of mitochondrial dynamics in the form of fission and fusion and for an obligatory physiological requirement of the processes. However, correlation rather than causality has been established. The experimental procedures on which the conclusion of an obligatory physiological requirement for mitochondrial dynamics relies on the manipulation of the fission and fusion proteins (e.g. Mfn, Opa1 and Drp1), not mitochondrial-shape or dynamics per se. Significantly, the proteins involved in mitochondrial fusion and fission (e.g. Mfn, Opa1 and Drp1) contribute to other cell activities that are critical for physiological function but unrelated to mitochondrial morphology transitions. For example, overexpression of Mfn2 suppresses, and downregulation of Mfn2 increases, vascular smooth muscle proliferation completely independently of the role of Mfn2 in mitochondrial-shaping activity [93]. Increased expression of Mfn2 triggers, and silencing Mfn2 protects against, vascular smooth muscle apoptosis, again in a way that is independent of mitochondrial fusion [94]. Mfn also tethers mitochondria and endoplasmic reticulum with significant consequences for Ca^2+^ signalling [95,96]. Mfn2 controls the expression of genes that encode proteins required for oxidative phosphorylation to occur (complexes I, II, III and V) via a mechanism that is completely unrelated to the protein's role in mitochondrial fusion [97]. Mfn2 downregulation represses the expression of nuclear-encoded subunits of complexes I, II, III and V and, presumably as a consequence, reduces the mitochondrial membrane potential [97]. Opa1 anchors A-kinase to lipid droplets to control perilipin phosphorylation and the breakdown of lipids [98], and can remodel mitochondrial cristae by a mechanism that is independent of the protein's role in fusion [99]. Drp1 localizes to peroxisomes and is essential for peroxisomal fission, a role independent of mitochondrial shaping [100]. Altering any of these processes by manipulating ‘mitochondrial-shaping proteins’ (Mfn, Opa1 and Drp1) could have significant physiological consequences, all of which are unrelated to mitochondria fission and fusion.

Indeed, at the cellular level, the precise physiological consequences that occur when fission and fusion are disrupted are uncertain. Mitochondrial fission and fusion have been linked to apoptosis and mitochondrial quality control via autophagy (mitophagy), but there is an abundance of contradictory data. For example, at an early stage in apoptosis, mitochondria fragment [101], which is an observation interpreted as mitochondrial fission contributing to apoptosis. Support is found in the observation that disrupting Drp1 may alter apoptosis [101,102]. Dominant-negative Drp1 and interfering RNA that is directed against Drp1 each prevent mitochondrial fragmentation during apoptosis and, interestingly, also reduce apoptosis [101]. However, in other studies, enhanced mitochondrial fission did not induce but rather protected against apoptosis [40,103,104]. Substantial Drp1 overexpression, and increased mitochondrial fission, induced neither necrotic nor apoptotic cell death and prevented Ca^2+^-dependent apoptosis [40].

Mitochondria fission may also be required to seg regate dysfunctional mitochondria for degradation by mitophagy [105]. The process may permit damaged mitochondria or mitochondria with mutated DNA to be removed. Indeed, Drp1 overexpression (resulting in fragmentation of mitochondria) promoted mitophagy, while overexpression of a dominant-negative form of Drp1 (Drp1^K38A^) prevented elimination of the organelle [105]. Additionally, Mfn1/2 must dissociate from mitochondria for mitophagy to proceed [106]. These observations suggest that dissociation of fusion proteins and increased fission must occur for mitophagy to progress. On the other hand, mitochondrial fission occurs normally in healthy cells without leading to mitophagy and a lack of mitochondrial fission may lead to mitochondrial oxidative damage, loss of mitochondrial DNA and increased mitophagy [107].

Thus, manipulating the proteins that are involved in the fission and fusion of mitochondria alters several cellular activities, but the precise consequences, and indeed the fundamental underlying physiological cause of the changes, are unclear. Part of the difficulty in unravelling the contribution of fission and fusion is that the proteins involved perform several functions that are unrelated to mitochondrial reshaping. Significantly, work at the cellular level has been almost exclusively on cultured cells and much less is known in native cells.

In conclusion, mitochondrial structural diversity arises from the organelle switching from being static to moving almost constantly or fusing and dividing in a dynamic, constantly reshaping network (fig. 5; online suppl. video 1). The processes that give rise to the changes in mitochondrial morphology, motion and shape interact closely to determine the mitochondrial arrangement (fig. 5). Mitochondrial dynamics are observed frequently in cultured cells and less so in native cell types. Nonetheless, in native vascular smooth muscle, significant changes in mitochondrial organisation occur during stress and proliferation. Indeed, it is clear that mitochondrial structure is controlled by cell activities. Conversely, while it is probable that the changing structure critically regulates several cell functions, precisely what role the various mitochondria arrangements perform in cell function is not understood. New methodologies to study mitochondrial phenotypes, particularly in native cells, are necessary to unravel the relationship between mitochondrial structure and function.

## Supplementary Material

suppl. video 1

suppl. video 2
